# Measurement invariance of the empowering and disempowering motivational climate questionnaire-coach in youth sport

**DOI:** 10.3389/fpsyg.2022.958444

**Published:** 2023-01-06

**Authors:** Paul R. Appleton, Carme Viladrich, Eleanor Quested, Lorena González-García, Athanasios Papaioannou, Howard K. Hall, Isabel Balaguer, Yago Ramis, Philippe Sarrazin, Jean-Philippe Heuzé, Yngvar Ommundsen, Bente Wold, Oddrun Samdal, Joan L. Duda

**Affiliations:** ^1^Department of Sport and Exercise Sciences, Musculoskeletal Science and Sports Medicine Research Centre, Faculty of Science and Engineering, Manchester Metropolitan University, Manchester, United Kingdom; ^2^Institute of Sport, Manchester Metropolitan University, Manchester, United Kingdom; ^3^Department of Psychobiology and Methodology of Health Sciences, Faculty of Psychology, Autonomous University of Barcelona, Barcelona, Spain; ^4^Curtin School of Population Health, Curtin University, Perth, WA, Australia; ^5^Physical Activity and Well-Being Research Group, enAble Institute, Curtin University, Perth, WA, Australia; ^6^Department of Social Psychology, Faculty of Psychology, University of Valencia, Valencia, Spain; ^7^Department of Physical Education and Sport Science, University of Thessaly, Trikala, Greece; ^8^Retired, Sutton on the Forest, York, United Kingdom; ^9^Department of Basic, Developmental and Educational Psychology, Faculty of Psychology, Autonomous University of Barcelona, Barcelona, Spain; ^10^Univ. Grenoble-Alpes, SENS, Grenoble, France; ^11^Department of Sport and Social Sciences, Norwegian School of Sport Sciences, Oslo, Norway; ^12^Department of Health Promotion and Development, Faculty of Psychology, University of Bergen, Bergen, Norway; ^13^School of Sport, Exercise and Rehabilitation Sciences, College of Life and Environmental Sciences, University of Birmingham, Birmingham, United Kingdom

**Keywords:** AGT, SDT, sport, youth, ESEM, invariance

## Abstract

The purpose of this study was to test the measurement invariance (across five languages, two time points, and two experimental conditions) of the empowering and disempowering motivational climate questionnaire-coach (EDMCQ-C; Appleton et al., 2016) when completed by 9256 young sport participants (*M* age = 11.53 years, *SD* = 1.39 years; 13.5% female). Exploratory Structural Equation Modeling was used to test the validity of a 2-factor (empowering and disempowering) model running a multiple group analysis without any equality constraint (configural invariance) followed by measurement invariance of factor loadings and thresholds (scalar invariance). Findings provided support for partial invariance across languages and scalar invariance across time and experimental groups. The factors were interpretable across the analyses, and items loaded as intended by theory except for item 15. This study provides further evidence regarding the psychometric properties of the EDMCQ-C and suggests this scale (minus item 15) can be used to provide meaningful latent mean comparisons (Marsh et al., 2013) of empowering and disempowering coach-created climates across athletes speaking the five targeted languages, across time, and across experimental groups.

## Introduction

For several decades, the coach-created motivational climate has been one of the most studied topics within sport psychology (see Duda et al., 2018). The motivational climate captures the social psychological environment that is created by significant others in sport, including coaches, and is relevant to variability in athletes’ cognitions, affect and behavior. This motivational environment concerns what the coach does, says and how he/she structures the environment in training and competitions (Duda, 2001).

Much of the work conducted on the coach-created motivational climate has been guided by achievement goal theory (AGT; Ames, 1992; Nicholls, 1989) and self-determination theory (SDT; Deci and Ryan, 1985, 2000). AGT and SDT identify specific facets of the motivational climate that have implications for athletes in and outside of sport. Whereas AGT places emphasis on task- and ego-involving climates, SDT recognizes dimensions of the climate that supports (e.g., autonomy-supportive, socially-supportive) or frustrates (e.g., controlling) an athlete’s psychological needs. Recently, Duda (2013) and Duda and Appleton (2016) proposed a multidimensional conceptualization of the coach-created motivational climate that integrates the social environmental dimensions forwarded by AGT and SDT. Duda’s framework considers that the coach-created motivational climate can be more or less ‘empowering’ and ‘disempowering’ in nature. Empowering climates are task-involving, autonomy-supportive and socially-supportive in nature and are theorized to be adaptive. Conversely, disempowering climates are ego-involving and controlling and have negative implications for athletes.

Within Duda’s conceptualization, empowering climates reflect adaptive coaching strategies emphasized by AGT and SDT. According to AGT, a task-involving climate is characterized by athletes perceiving that trying hard, skill development and cooperative learning between teammates are valued by the coach (Newton et al., 2000). Within SDT, an autonomy-supportive climate includes athletes’ preferences being recognized and their perspectives considered, their feelings are acknowledged, they are provided with meaningful choices, their input into decision-making (when and where possible) is welcomed, and a rationale is provided when they are asked to do something by the coach (Mageau and Vallerand, 2003). In a socially-supportive environment, every athlete feels cared for and is empathized with, and is valued as an athlete and as a person (Mageau and Vallerand, 2003; Reinboth et al., 2004).

In contrast, disempowering climates are marked by the negative and debilitating coaching strategies recognized in AGT and SDT. An ego-involving climate, for example, is characterized by athletes perceiving that mistakes result in punishment, the coach providing differential treatment based on the ability level of athletes, and that intra-team member rivalry is encouraged on the team (Newton et al., 2000). Relatedly, a controlling climate is evident when coaches pressure, coerce and intimidate their athletes (Bartholomew et al., 2010).

To enable researchers to measure empowering and disempowering motivational climates, Appleton et al. (2016) developed the 34-item Empowering and Disempowering Motivational Climate Questionnaire-Coach (EDMCQ-C) using data from samples of young British sport participants. The theoretical model proposed by Duda (2013; Duda and Appleton, 2016) that informed the development of the EDMCQ-C includes five lower order factors (task- and ego-involving, autonomy- and social-supportive, and controlling) and two higher-order dimensions (empowering and disempowering). Moreover, although it is possible to capture data on the motivational climate using a range of distinct albeit related sources (e.g., coach self-report; objective observations), the EDMCQ was originally developed by Appleton et al. to measure athletes’ perceptions of their coaches’ empowering and disempowering strategies in training and competition. Capturing athletes’ views of empowering and disempowering features of the coach-created motivational climate is important given they are the most proximal predictor to variability in athletes’ cognitions, affect and behaviors. Furthermore, compared to coaches’ self-reports of their motivational climate, athletes’ own reports tend to be closer to reality (i.e., what coaches are actually doing; Smith et al., 2016).

The 34 items included in the EDMCQ-C were identified from a pool of 67 statements from established measures of the five lower order climate factors using confirmatory factor analyses. Appleton et al. (2016) subsequently tested a series of factor structures using exploratory structural equation models (ESEM). ESEM integrates the principles of Exploratory Factor Analysis (i.e., items permitted to load on intended factor and crossload on non-intended factors) within the SEM framework (i.e., fit indices to assess model fit) (Asparouhov and Muthén, 2009). The first ESEM model tested by Appleton et al. was a lower-order five factor (task, autonomy-supportive, socially-supportive, controlling, ego) model. The second model tested the proposed hierarchical structure of the EDMCQ-C using Hierarchical ESEM (HESEM) in which the aforementioned lower-order dimensions were modeled onto their respective higher order factor (i.e., task, autonomy-supportive, socially-supportive loaded on an empowering higher-order dimension; control and ego loaded on a disempowering higher-order dimension). Finally, Appleton et al. also tested a bi-factor ESEM (BESEM) which is represented by two higher-order (or “general”) factors (e.g., empowering and disempowering climates), five lower-order (or “group”) factors (e.g., task- and ego-involving climates, autonomy- and social-supportive climates, and controlling climates), and a pattern matrix in which items loads onto the general and group factors.

All ESEM models resulted in acceptable model fit (e.g., CFI > 0.94; TFI > 0.91; RMSEA ≤ 0.30), with the better fit achieved *via* the BESEM model. However, across all ESEM models, inspection of the standardized factor loadings revealed that the majority of autonomy-supportive and some controlling and socially-supportive items failed to load significantly on their intended factor and demonstrated elevated and significant factor loadings on their non-intended climate dimension. More specifically, autonomy- and socially-supportive items loaded onto the task-involving dimension and controlling items loaded significantly onto the ego-involving factor.

In sum, the findings reported by Appleton et al. (2016) do not lend support for the multidimensional, hierarchical structure proposed to underpin the EDMCQ-C. Rather than abandon the scale, however, researchers have proposed that the scale’s structure may be best represented by two main climate factors (empowering and disempowering). This is evident in the Appleton et al. (2016) study where many of the items from the empowering lower-order dimensions (task, autonomy-supportive, socially-supportive) loaded significantly onto one factor, and many items from the disempowering lower-order dimensions (control, ego) loaded onto a second factor. This two-factor model has also been tested using ESEM (in which the 34-items are permitted to load on both factors) in subsequent research (Milton et al., 2018; Solstad et al., 2020; Sukys et al., 2020).

Although adopting a two-factor model prevents researchers from using the scale to examine the multidimensional and hierarchical structure of the climate as emphasized in Duda’s framework, adopting a two-factor approach to modeling the EDMCQ-C would still permit researchers the opportunity to examine empowering and disempowering coach-created motivational climates and examine their association with athletes’ cognitions, affect and behavior (e.g., Appleton and Duda, 2016; Smith et al., 2016; Fenton et al., 2017). In addition, this simpler two-factor model (in comparison to the HESEM and BESEM models tested by Appleton et al., 2016) would reduce the complexities associated with establishing the psychometric properties (e.g., invariance) of the scale (Milton et al., 2018; Solstad et al., 2020).

Adopting the two-factor structure supported in previous research, the main purpose of this study was to further contribute to evidence regarding the psychometric properties of the EDMCQ. This was achieved by using ESEM to test measurement invariance using data from young football players from five European countries (i.e., France, Greece, Norway, Spain, and England) who spoke the main language in each country (e.g., English in England; Spanish in Spain etc.), across time (start and end of season), and between experimental conditions (intervention and control groups).

## Materials and methods

### Participants

The data come from the large European-wide PAPA project (see Duda et al., 2013) that sought to promote healthy and sustained physical activity in children and adolescents *via* participation in grassroots football. Player questionnaire data from the PAPA project on the motivational climate has been used in other studies, albeit limited to Time 1 data in England (Appleton et al., 2016) or one facet of the climate (i.e., autonomy-support) in five countries (Quested et al., 2013). 9256 football players (13.5% female) aged between 9 and 15 years (mean = 11.53, SD = 1.39) from five European countries (France = 1426, Greece = 1707, Norway = 1998, Spain = 2335, and the England = 1790) participated in this study. The participants played for 638 teams at the grassroots level, reported to have been playing for their teams between the present season and the last 10 seasons (median = 3 seasons, interquartile range = 4), and trained between 0.5 and 10 h a week (median = 4, interquartilerange = 2). 61.4% of the participants were from the intervention group in the PAPA project. The intervention (*Empowering Coaching*™; see Duda, 2013) involved the participants’ coaches attending a 6-hour education workshop concerning the creation of empowering (and less disempowering) motivational climates in training and matches. The coaches of the participants in the control condition engaged in their normal coaching practices. See Table 1 for an overview of participants’ demographic information by country.

**TABLE 1:** Participants’ demographic information by country.

	England	France	Spain	Norway	Greece
**Gender**					
Boy	1553	1390	2117	1254	1649
Girl	237	36	213	744	22
**Condition**					
Experimental	1019	895	1235	1452	1082
Control	771	531	1100	546	625
**Age**					
9–12 years	1019	859	1684	940	1000
13–15 years	343	353	544	431	462
Number of seasons on team (*M*, *SD*)	2.5 (1.9)	3.3 (2.4)	3.2 (2.2)	4.4 (2.2)	3.1 (2)
Number of hours per week playing with team (*M*, *SD*)	2.8 (1.1)	4.7 (1.1)	4.7 (1.2)	3.5 (1.7)	4.8 (1.7)
Number of hours per week spent with coach (*M*, *SD*)	2.9 (1.1)	4.7 (1.1)	4.7 (1.3)	N/A	4.9 (1.7)

N/A, not applicable.

### Procedures

The protocol outlining the procedures associated with data collection in the PAPA project were described in Duda et al. (2013) and employed in previous studies (e.g., Quested et al., 2013). Ethical approval was granted by the Universities of each team of researchers working on the PAPA project. Prior to collecting data, parents had the opportunity to opt out their son and/or daughter from the project. Participants were fully informed about the study before it took place, and a parental written opt out form was used in accordance with national legislation and the institutional requirements.

The EDMCQ-C was one of a number of scales included in the overall player questionnaire employed in the PAPA project. Time 1 data was collected at the start of the competitive football season (spring 2011 in Norway and between autumn and winter 2011/2012 in the other countries). Time 2 data was collected at the end of the same season, approximately 20–28 weeks after Time 1 (in late summer 2011 in Norway, and spring 2012 in the other countries). Data was collected by trained research assistants working on the PAPA project. Each participants completed the questionnaire individually albeit were grouped with their teammates typically during a training session. The overall questionnaire took between 20–40 min to complete, and the EDMCQ-C took between 5–10 min (see Duda et al., 2013 for further details).

### Measure

All text in the questionnaire was initially drafted in English, translated into Spanish, Norwegian, Greek, and French by a native speaker, and then back-translated into English by a second dual-language speaker. The translation-back translation procedure was based on the recommendations from mainstream (Harkness, 1999; Hambleton, 2005) and sport psychology literature (Duda and Hayashi, 1998).

The EDMCQ-C (Appleton et al., 2016) includes 17 empowering items measuring task-involving (e.g., “My coach encouraged athletes to try new skills”), autonomy-supportive (e.g., “My coach gave athletes choices and options”) and socially-supportive (e.g., “My coach really appreciated athletes as people, not just as a sport participants”) coaching. 17 disempowering items are also included measuring ego-involving (e.g., “My coach yelled at athletes for messing up”) and controlling (e.g., “My coach paid less attention to athletes if they displeased him or her”) climate dimensions. Participants were instructed to “think about what it has usually been like on *this* team/club *during the last 3–4 weeks*” when providing their responses, which were measured on a 5-point scale (i.e., 1 = *strongly disagree*, 5 = *strongly agree*).

### Data management and analysis

The data were validated and screened for patterns of missing values prior to the creation of an international file for main analysis. Although the majority of the sample (9194 cases) responded to the EDMCQ-C at Time 1 and/or Time 2, only 35% of the sample had complete data for all items and both time points. To detect any bias attributable to data missingness, we analyzed the data twice: first using the whole sample and second with the subsample that provided complete data. As the results of both analyses were similar, only the results obtained using the whole sample are presented.

To test for measurement invariance, target rotation (Asparouhov and Muthén, 2009; Marsh et al., 2013) in the ESEMs conducted in Mplus (Muthén and Muthén, 1998–2015) were employed. A target rotation consists of defining which factor loadings will be freely estimated (those for items on their intended factor) and which factor loadings will be restricted to values as close as possible to zero (those for items on their non-intended factor/s; Muthén, and Muthén, 1998-2017). In our study, task-involving, autonomy-supportive and socially-supportive items were freely estimated on an empowering factor and permitted to cross-load on the disempowering factor but with factor loadings restricted to values close as possible to zero. Controlling and ego involving items were freely estimated on the disempowering factor and permitted to cross-load on the empowering factor but with factor loadings restricted to values close as possible to zero. We followed the recommendations proposed by Millsap and Yun-Tein (2004) for categorical variables, and Marsh et al. (2013) and Marsh et al. (2020) for ESEMs. First, we tested the validity of a 2-factor model in each country using ESEM running a multiple group analysis without any equality constraint (configural invariance). We then tested measurement invariance of factor loadings and thresholds (scalar invariance; Muthén and Muthén, 1998–2015; Wang and Su, 2013) across countries, time, and experimental condition in separate steps. Total or partial scalar invariance of items ensures meaningful latent mean comparisons across groups and over time (e.g., Marsh et al., 2013).

Due to the complexity of the analyses (5 countries * 2 conditions * 2 waves), we did not employ the “interactional” tests of invariance outlined in Grouzet et al. (2006). Moreover, we did not have reason to test invariance in any specific order (Wang and Su, 2013). For the test of invariance across time, we correlated the error terms from the same items on different occasions to account for within subjects’ data (Marsh and Hau, 1996). In all analyses, factor loadings and thresholds were constrained to be equal in tandem as it is recommended for categorical-ordered indicators (Muthén and Muthén, 1998–2015).

Due to the categorical nature of the data and the presence of missing values (see Results section), the weighted least-squares mean and variance-adjusted estimator was used with pairwise deletion for missing values, both of them being the Mplus defaults for categorical data. The Goodness-of-fit Indices were χ2, Comparative Fit Index (CFI), Tucker–Lewis Index (TLI), and root mean square error of approximation (RMSEA). In an independent clusters confirmatory factor analysis model with quantitative indicators, CFI and TLI values >0.95 and RMSEA <0.06 are considered as indicators of excellent fit (Hu and Bentler, 1995), and CFI and TLI values >0.90 and RMSEA <0.08 are considered as indicators of acceptable fit (Marsh et al., 2004). Little simulation data are available on the behavior of these cut-off values in categorical data ESEM analysis, but Yu (2002) suggested using a CFI >0.96 for categorical data and most papers using ESEM, including categorical data ESEM (Myers et al., 2011), rely on them with some caution (but see Maydeu-Olivares et al., 2017). Standard errors and fit indices were calculated taking into account that players’ responses were clustered within their teams.

In order to compare nested models, we employed *ad hoc* guidelines to evaluate differences in fit, including the difference in CFI (ΔCFI) and RMSEA (ΔRMSEA). As a cut-off value, a ΔCFI < 0.01 (Cheung and Rensvold, 2002) and changes in RMSEA <0.015 (Chen, 2007) are considered as evidence for the more parsimonious model. Marsh (2007) also proposed that the more parsimonious models is supported if the TLI and RMSEA are as good as or better than that for the more complex model. However, Marsh also proposed that these above proposals for assessing invariance should be considered as rough guidelines rather than golden rules. All these recommendations were considered in this paper.

Regarding reliability, we followed the advice of Viladrich et al. (2017) regarding the coefficient H, also known as maximal reliability (Raykov, 2012), that should be used to get an adequate reliability estimate when the intended measures are factors instead of unweighted composite measures. As H is strongly related to the size of factor loadings but not directly applicable to ordinal data, we used the size of the factor loadings to gauge the reliability of the EMP and DISEMP factor scores.

## Results

Responses to the questionnaire showed sizeable floor (disempowering items) or ceiling (empowering items) effects (see Table 2), confirming the decision to treat the data as categorical using the WLSMV estimator in MPlus. As reported in Table 2, the most frequent response in each country was “agree” (16–52% of responses) and “strongly agree” (17–72% of responses) for the majority of the empowering items at both time points, with fewer participants responding “strongly disagree” (1–12% of responses) or “disagree” (1–13% of responses). One exception was empowering item three “My coach offers choices and options”, where the proportion of the French sample was similar across the five response categories. For the disempowering items, the proportion of the sample was higher for the “strongly disagree” (10–57% of responses), “disagree” (11–39% of responses) and “neutral” (13–38% of responses) responses across the countries and at each time point with fewer participants responding “strongly agree” (1–7% of responses) or “agree” (5–33% of responses), albeit there were some exceptions. For example, for disempowering item 15 “My coach only allowed something we like to do at the end of training if players had done well during the session,” there was a larger proportion of the sample who responded “agree” and fewer who responded “strongly disagree” compared to the other disempowering items.

**TABLE 2:** Percentage of responses to each category by time and language.

Time1	Strongly disagree	Disagree	Neutral	Agree	Strongly agree
**Items**					
E1	1/2/2/1/8	2/3/3/3/10	12/17/16/15/24	52/39/48/51/30	33/39/31/31/28
E4	1/2/2/2/3	1/2/3/2/4	7/6/13/13/13	35/20/40/42/27	56/72/42/42/53
E11	1/2/2/2/3	2/3/3/4/3	10/12/15/20/14	44/31/35/42/30	43/53/45/33/50
E13	2/3/10/3/6	2/2/11/4/7	16/8/29/9/24	43/27/29/36/30	37/61/20/48/33
E18	2/2/1/2/5	2/4/3/4/4	20/23/20/26/19	49/30/48/46/34	27/41/29/22/38
E23	2/4/1/2/3	3/4/3/3/5	13/16/18/14/15	44/27/48/42/31	39/49/29/39/46
E28	2/3/1/2/5	2/4/2/3/5	14/15/13/15/23	39/24/33/38/29	43/55/51/42/38
E30	2/3/1/2/4	3/4/3/4/4	16/14/17/16/14	43/25/44/39/32	36/54/35/39/45
E34	1/2/1/2/3	2/2/1/2/3	11/7/8/7/10	28/16/32/27/28	58/72/59/61/57
E3	2/5/4/4/22	4/4/10/7/16	18/21/32/20/28	50/35/36/43/22	26/35/18/26/12
E6	2/4/1/2/5	3/3/2/5/5	22/14/13/16/11	44/25/34/37/22	30/55/50/41/57
E16	1/4/1/2/3	4/4/4/4/6	23/13/26/12/20	42/28/43/39/32	31/51/26/43/39
E22	2/2/2/2/3	4/3/4/4/5	26/15/26/13/17	45/26/41/42/33	24/54/27/40/42
E32	2/2/2/3/8	3/3/3/5/6	18/12/14/19/17	38/19/35/40/24	39/63/47/34/45
E8	2/2/2/3/4	3/3/4/5/4	23/9/19/19/20	40/20/39/40/32	32/67/37/33/40
E14	2/4/2/3/5	3/4/3/5/5	19/19/25/17/30	43/25/37/37/28	0.33/49/34/38/33
E27	9/10/2/7/9	9/9/3/10/9	24/28/26/31/36	33/21/40/30/22	25/32/30/22/23
D5	33/29/45/22/31	35/18/27/27/17	20/28/21/29/23	9/13/5/14/16	3/12/3/8/13
D9	38/51/26/39/43	35/19/28/32/24	17/16/29/16/17	7/8/12/8/8	4/6/6/6/8
D10	36/40/52/9/28	27/20/25/17/17	23/21/17/31/22	10/9/4/28/20	5/10/2/15/13
D19	31/31/27/30/45	27/16/24/25/19	24/27/27/27/20	11/12/14/12/7	7/14/8/6/9
D21	18/32/37/22/36	31/18/31/29/22	30/22/22/29/19	15/14/7/15/13	7/14/3/5/11
D25	37/50/24/43/53	30/18/20/29/16	19/18/25/16/16	9/6/19/8/9	4/1/12/5/6
D33	34/41/28/30/36	27/19/23/30/20	24/21/29/25/23	11/10/13/11/11	5/9/8/4/10
D2	21/40/28/24/42	30/20/30/32/20	26/24/29/25/21	17/0.10/10/15/11	5/6/4/5/8
D7	24/39/33/29/42	33/19/26/31/19	28/23/28/21/21	11/11/10/15/9	4/9/4/5/8
D12	26/39/32/29/43	36/24/28/34/25	27/24/30/23/28	8/8/8/10/7	3/5/3/4/6
D15	6/16/7/17/33	10/11/9/25/12	36/29/31/30/25	29/19/34/18/24	19/25/21/11/23
D17	23/36/40/24/26	31/21/34/37/22	33/27/20/28/28	10/10/5/8/14	3/6/2/3/11
D20	23/55/7/48/56	22/15/11/26/20	31/16/36/16/13	15/7/31/6/6	10/7/15/4/5
D24	25/23/40/23/24	27/15/26/25/15	28/25/2/27/25	14/18/10/17/20	6/19/3/9/16
D26	47/37/53/31/30	22/15/24/22/14	16/20/14/22/20	11/14/7/17/18	4/15/3/8/17
D29	12/36/27/33/29	19/14/32/26/17	38/23/30/23/29	19/14/7/13/13	13/13/4/6/11
D31	35/57/35/21/37	23/15/33/21/16	26/16/25/32/27	10/6/5/18/11	6/6/2/9/9
**Time 2**					
**Items**					
E1	2/3/2/2/11	3/4/3/3/13	13/17/18/14/28	49/35/45/49/30	33/42/33/32/17
E4	1/2/1/1/5	2/3/4/3/5	7/8/13/18/16	33/24/43/42/34	56/63/40/37/40
E11	1/3/2/2/4	2/4/2/5/6	11/17/12/20/17	46/32/40/46/34	40/45/44/27/39
E13	12/3/10/2/5	3/5/11/3/7	18/14/28/12/24	44/31/29/42/35	34/48/23/40/29
E18	1/3/1/1/6	4/5/3/5/6	21/27/16/26/21	47/30/51/45/36	27/35/30/23/31
E23	2/3/1/10/5	4/8/3/5/5	21/17/18/17/19	41/30/47/44/35	32/42/31/34/37
E28	2/3/1/1/5	4/5/3/4/6	20/19/14/15/25	35/27/34/40/33	39/45/48/39/31
E30	2/4/1/2/6	5/7/3/5/4	21/17/17/18/22	40/28/45/40/37	32/45/34/36/31
E34	1/2/0/1/4	1/4/2/2/4	11/11/9/12/15	35/23/33/34/34	52/61/57/52/44
E3	2/4/3/4/29	5/6/8/6/21	18/25/31/22/26	50/35/38/45/17	25/30/20/22/7
E6	1/4/2/1/4	3/4/1/4/4	25/17/13/18/12	42/26/32/43/27	28/50/52/33/52
E16	2/3/1/2/4	5/7/5/3/6	24/17/24/15/21	43/30/47/43/37	27/44/24/38/32
E22	2/3/2/1/4	6/5/1/5/8	3/19/13/16/21	41/30/32/46/37	22/43/52/32/31
E32	1/3/1/2/7	4./5/2/4/7	18/15/14/19/18	40/27/33/39/37	38/52/50/37/39
E8	2/2/2/3/6	4/5/3/6/7	22/13/19/23/24	38/24/41/37/32	33/55/36/31/31
E14	1/4/2/2/5	4/6/3/5/7	18/23/21/19/33	42/27/37/36/31	40/40/37/38/25
E27	7/9/2/7/6	11/11/4/10/7	29/28/25/29/26	31/24/40/33/33	22/28/30/22/28
D5	33/21/41/23/26	32/22/28/27/19	23/31/22/31/27	7/15/6/13/18	5/11/3/7/11
D9	35/41/22/34/40	37/21/26/33/25	16/18/30/19/20	8/12/14/10/8	4/8/8/4/7
D10	32/33/49/10/22	31/24/26/19/20	23/23/19/33/24	10/10/4/29/21	4/9/2/10/13
D19	31/28/25/25/45	26/18/21/25/20	27/27/29/29/17	10/13/15/14/8	7/14/11/7/10
D21	20/28/35/23/30	27/20/29/29/22	31/25/23/30/24	17/16/9/13/14	6/12/5/5/10
D25	32/40/19/41/46	29/21/19/29/18	26/22/24/17/18	09/10/22/9/11	4/7/15/4/07
D33	31/31/24/27/32	30/22/21/30/21	23/22/29/23/23	11/14/16/14/14	5/11/11/7/11
D2	26/38/29/28/40	27/23/28/34/24	28/24/29/25/21	14/9/10/11/9	5/6/5/3/6
D7	25/33/33/27/37	32/22/26/31/24	28/25/26/27/24	12/12/11/13/8	4/8/5/2/7
D12	27/33/31/24/31	39/27/30/39/26	25/24/27/24/30	7/10/9/10/8	4/6/3/3/5
D15	5/3/6/16/15	12/11/9/27/14	36/28/29/33/31	30/23/35/17/25	17/24/24/7/16
D17	21/31/39/27/22	33/22/33/34/25	34/29/22/28/29	9/11/4/9/16	3/7/2/2/8
D20	24/52/6/45/49	27/16/11/25/21	29/17/33/18/18	14/10/33/7/7	6/6/17/5/5
D24	27/20/39/23/21	25/18/26/23/20	28/28/20/32/28	14/19/11/15/17	5/14/5/7/15
D26	40/33/53/30/28	24/17/21/22/16	20/23/13/24/21	11/14/9/18/20	5/13/4/7/16
D29	14/28/27/31/27	20/18/32/30/19	41/28/30/23/33	17/15/9/11/12	8/11/4/5/10
D31	34/45/36/20/31	23/18/34/21/21	28/20/23/35/28	10/9/5/16/12	5/9/3/8/8

NBE, Empowering item; D, Disempowering item; English/Spanish/Norwegian/Greek/French.

### Language invariance

At Time 1, indexes of fit for configural invariance across languages were: χ^2^(2470) = 5932.68, CFI = 0.952, TLI = 0.945, RMSEA = 0.030 (CI 95% = 0.029–0.031) and scalar invariance χ^2^(3126) = 10644.48, CFI = 0.895, TLI = 0.906, RMSEA = 0.039 (CI 95% = 0.039–0.042). Results were similar for Time 2: indexes of fit for configural invariance were: χ^2^(2470) = 5908.73, CFI = 0.953, TLI = 0.946, RMSEA = 0.034 (CI95% = 0.032–0.035) and scalar invariance χ^2^(3126) = 9362.04, CFI = 0.914, TLI = 0.923, RMSEA = 0.040 (CI95% = 0.039–0.041). Although ΔRMSEA met the adopted criteria of <0.015 (Chen, 2007), ΔCFIs were > 0.01 and TLI and RMSEA values were not as good or better for scalar invariance compared to configural invariance (see Table 3). Inspection of the modification indices revealed non-invariant thresholds for a number of items. We therefore freed the thresholds of these items in partially invariant models. At Time 1, indexes of fit for partial invariant model were χ^2^(3086) = 8950.36, CFI = 0.918, TLI = 0.926, RMSEA = 0.035 (CI95% = 0.034–0.036) and Time 2 χ^2^(3086) = 7966.11, CFI = 0.933, TLI = 0.939, RMSEA = 0.036 (CI95% = 0.035–0.037) (see Table 3).

**TABLE 3:** Model fit of the approximate invariance test across language, time and condition.

Model	χ2	df	CFI	TLI	RMSEA	RMSEA 90% CI
***Language Time 1***						
Configural invariance	5932.68	2470	0.952	0.945	0.030	0.029–0.031
Scalar invariance	10644.48	3126	0.895	0.906	0.039	0.039–0.042
Partial invariance	8950.36	3086	0.918	0.926	0.035	0.034–0.036
***Language Time 2***						
Configural invariance	5908.73	2470	0.953	0.946	0.034	0.032–0.035
Scalar invariance	9362.04	3126	0.914	0.923	0.040	0.039–0.041
Partial invariance	7966.11	3086	0.933	0.939	0.036	0.035–0.037
***Time***						
Configural invariance	8982.89	2106	0.937	0.931	0.019	0.018–0.019
Scalar invariance	10229.91	2307	0.927	0.928	0.019	0.019–0.020
***Condition Time 1***						
Configural invariance	5497.15*	988	0.925	0.915	0.034	0.033–0.035
Scalar invariance	4465.67*	1152	0.945	0.947	0.027	0.026–0.028
***Condition Time 2***						
Configural invariance	5656.37*	988	0.924	0.913	0.039	0.038–0.040
Scalar invariance	4195.22*	1152	0.950	0.952	0.029	0.028–0.030

df, degrees of freedom; CFI, comparative fit index; TLI, Tucker–Lewis index; RMSEA, root mean square error of approximation; CI, confidence interval.

**FIGURE 1:**
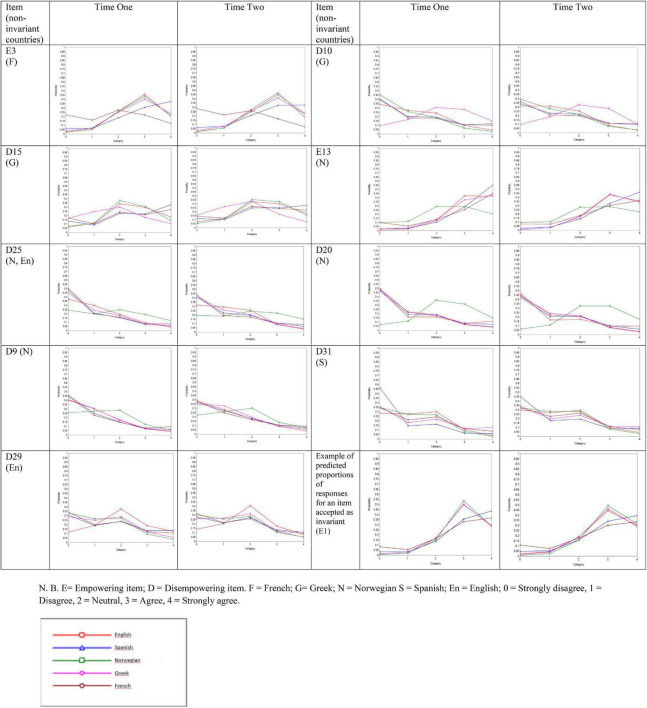
Predicted proportions of responses for non-invariant items from the empowering and disempowering motivational climate questionnaire-coach (EDMCQ-C). NBE, empowering item; D, disempowering item. F, French; G, Greek; N, Norwegian; S, Spanish; En, English; 0, strongly disagree; 1, disagree; 2, neutral; 3, agree; 4, strongly agree.

Non-invariant thresholds emerged for nine items (see Figure 1). Empowering item three was non-invariant in French with predicted proportions of responses more uniformly distributed across all categories compared to the other countries with distributions relatively skewed towards agreeing with this item. Disempowering items 10 and 15 were both more uniformly distributed for Greek than for the other countries. Empowering item 13 and disempowering items nine, 20 and 25 were more uniformly distributed in Norwegian. Disempowering items 25 and 29 were relatively more uniformly distributed in English. As an exception, item 31 in Spanish was non-invariant due to a predicted distribution of responses more skewed toward strongly disagree responses compared to the other languages.

Non-standardized factor loadings were statistically invariant across languages, thus only factor loadings for the reference language (i.e., English) are reported (see Table 4) in a standardized form. They revealed all empowering and disempowering items loaded positively and more strongly on their intended factor than on the non-intended factor at times 1 and 2 except for disempowering item 15 (“My coach only allowed something we like to do at the end of training if players had done well during the session”) which loaded more strongly onto the empowering factor in all countries. For the non-reference languages (i.e., Spanish, Norwegian, Greek, French), standardized factor loadings showed no major discrepancies (around 0.10) in each factor loading, except for autonomy-supportive item one (“My coach gave players choices and options”) in French with standardized factor loadings of 0.15 at Time 1 and 0.10 at Time 2. Both values were lower than those of the other countries which ranged from 0.40 to 0.57 at Time 1 and from 0.46 to 0.61 at Time 2. Overall, the majority of factor loadings were high (>0.50) suggesting indirect evidence for internal consistency (reliability) for the empowering and disempowering latent variables across the five countries.

**TABLE 4:** Standardized factor loadings for the 34 items from the empowering and disempowering motivational climate questionnaire-coach (EDMCQ-C) across language, time, and condition.

	Language invariance^a^				Time invariance^b^		Condition invariance^c^			
Items	EmT1	DisT1	EmT2	DisT2	Em	Dis	EmT1	DisT1	EmT2	DisT2
E1	**0.54**	–0.03	**0.59**	–0.05	0.56	–0.01	**0.51**	–0.02	**0.63**	0.00
E4	**0.64**	–0.07	**0.65**	–0.10	**0.64**	–0.05	**0.62**	–0.05	**0.65**	–0.06
E11	**0.70**	0.00	**0.72**	0.00	**0.63**	0.02	**0.62**	0.01	**0.66**	0.00
E13	**0.67**	0.21	**0.76**	0.27	**0.56**	0.23	**0.59**	0.21	**0.61**	0.19
E18	**0.66**	0.00	**0.70**	0.00	**0.63**	0.02	**0.61**	0.00	**0.68**	0.01
E23	**0.70**	–0.03	**0.69**	–0.05	**0.66**	–0.03	**0.65**	–0.03	**0.69**	–0.04
E28	**0.66**	–0.08	**0.67**	–0.07	**0.64**	–0.07	**0.64**	–0.09	**0.68**	–0.05
E30	**0.70**	–0.03	**0.70**	–0.02	**0.66**	–0.02	**0.65**	–0.03	**0.67**	–0.03
E34	**0.64**	–0.07	**0.69**	–0.04	**0.67**	–0.04	**0.63**	–0.06	**0.70**	–0.02
E3	**0.54**	0.08	**0.61**	0.03	**0.47**	0.09	**0.44**	0.06	**0.53**	0.08
E6	**0.54**	0.01	**0.52**	–0.03	**0.51**	0.00	**0.50**	0.02	**0.55**	–0.04
E16	**0.64**	–0.07	**0.66**	–0.03	**0.60**	–0.05	**0.56**	–0.06	**0.62**	–0.05
E22	**0.63**	0.05	**0.65**	0.06	**0.65**	0.05	**0.62**	0.04	**0.67**	0.04
E32	**0.65**	0.00	**0.63**	–0.03	**0.63**	0.00	**0.65**	0.00	**0.67**	–0.03
E8	**0.57**	–0.13	**0.61**	–0.14	**0.61**	–0.12	**0.57**	–0.12	**0.64**	–0.13
E14	**0.64**	–0.05	**0.67**	–0.03	**0.58**	–0.02	**0.57**	–0.03	**0.63**	–0.03
E27	**0.37**	–0.02	**0.47**	–0.02	**0.46**	–0.03	**0.41**	–0.03	**0.53**	–0.02
D5	0.07	**0.48**	0.02	**0.58**	0.08	**0.55**	0.10	**0.48**	0.02	**0.53**
D9	–0.10	**0.65**	–0.09	**0.67**	–0.10	**0.63**	–0.12	**0.58**	–0.09	**0.65**
D10	0.02	**0.67**	–0.04	**0.69**	0.00	**0.60**	0.03	**0.57**	–0.07	**0.56**
D19	0.00	**0.64**	0.02	**0.73**	0.04	**0.64**	0.01	**0.59**	0.05	**0.70**
D21	0.00	**0.58**	0.00	**0.61**	0.04	**0.58**	0.03	**0.48**	0.02	**0.58**
D25	–0.13	**0.60**	–0.04	**0.66**	–0.10	**0.56**	–0.16	**0.53**	–0.04	**0.62**
D33	–0.11	**0.67**	–0.03	**0.72**	–0.09	**0.67**	–0.12	**0.63**	–0.07	**0.73**
D2	–0.01	**0.57**	–0.03	**0.55**	–0.01	**0.51**	0.00	**0.44**	–0.03	**0.53**
D7	–0.04	**0.54**	–0.08	**0.60**	–0.02	**0.55**	–0.01	**0.49**	–0.06	**0.58**
D12	.-09	**0.58**	–0.10	**0.68**	–0.09	**0.58**	–0.01	**0.55**	–0.11	**0.60**
D15	0.41	**0.20**	0.39	**0.18**	0.36	**0.20**	0.35	**0.18**	0.40	**0.20**
D17	–0.08	**0.62**	–0.07	**0.69**	–0.06	**0.61**	–0.06	**0.58**	–0.07	**0.61**
D20	0.08	**0.35**	0.04	**0.39**	0.06	**0.35**	0.06	**0.35**	0.08	**0.39**
D24	0.08	**0.69**	0.11	**0.76**	0.14	**0.63**	0.12	**0.55**	0.09	**0.58**
D26	0.00	**0.63**	0.05	**0.65**	0.05	**0.53**	0.03	**0.48**	0.04	**0.52**
D29	0.23	**0.38**	0.08	**0.18**	0.18	**0.35**	0.19	**0.33**	0.16	**0.33**
D31	–0.02	**0.40**	–0.01	**0.36**	–0.05	**0.35**	–0.07	**0.36**	–0.04	**0.36**

Em, empowering climate; Dis, disempowering climate; T, time; ^a^standardized factor loadings of reference group (England); ^b^standardized factor loadings of time one; ^c^standardized factor loadings of reference group (control). The bold values indicate the items that are expected to load on the specified factor.

Finally, the correlation between the empowering and disempowering factor at Time 1 was –0.48 (*p* < 0.001) in English, –0.67 (*p* < 0.001) in Spanish, –0.68 (*p* < 0.001) in Norwegian, –0.28 (*p* < 0.001) in Greek, and –0.36 (*p* < 0.001) in French. At Time 2, the correlation was –0.58 (*p* < 0.001) in English, –0.82 (*p* < 0.001) in Spanish, –0.66 (*p* < 0.001) in Norwegian, –0.48 (*p* < 0.001) in Greek, and –0.67 (*p* < 0.001) in French.

### Time invariance

Indexes of fit for configural invariance were χ^2^(2106) = 8982.89, CFI = 0.937, TLI = 0.931, RMSEA = 0.019 (CI95% = 0.018–0.019) and scalar invariance χ^2^(2307) = 10229.91, CFI = 0.927, TLI = 0.928, RMSEA = 0.019 (CI95% = 0.019–0.020). ΔCFI = 0.01, ΔRMSEA <0.015 and TLI and RMSEA values were as good for scalar invariance compared to configural invariance, offering support for scalar invariance (see Table 3).

Non-standardized factor loadings were statistically invariant across time, thus only factor loadings for Time 1 are reported in standardized form. Inspection of the factor structure revealed that empowering and disempowering factors were clearly distinguishable, with 33 items loading ranging from 0.35 to 0.67 (*p* < 0.001) on the intended factor (see Table 4). Standardized factor loadings of the non-intended factor ranged from –0.14 to 0.38. As per the language analyses, item 15 loaded more strongly on the empowering factor (0.383) than disempowering factor (0.206). However, as the majority of factor loadings were high (>0.50) for items on their intended factor, there is indirect evidence for internal consistency (reliability) for the empowering and disempowering latent variables across time. The correlations between the error terms from the same items on different occasions ranged from 0.04 to 0.38.

The correlation between the empowering climate factors at Time 1 and Time 2 was 0.52 (*p* < 0.001), and between disempowering climate factors at Time 1 and Time 2,0.55 (*p* < 0.001). The correlation between the empowering and disempowering factors was –0.51 (*p* < 0.001) at Time 1 and –0.55 (*p* < 0.001) at Time 2. The correlation between empowering factor at Time 1 and disempowering factor at Time 2 was –0.30 (*p* < 0.001). Finally, the correlation between the disempowering factor at Time 1 and the empowering factor at Time 2 was –0.34 (*p* < 0.001).

### Condition invariance

Indexes of fit for configural invariance across condition at Time 1 one were χ^2^(988) = 5997.15, CFI = 0.925, TLI = 0.915, RMSEA = 0.034 (CI95% = 0.033–0.035) and scalar invariance χ^2^(1152) = 4465.67, CFI = 0.945, TLI = 0.947, RMSEA = 0.027 (CI95% = 0.026–0.028). ΔCFI = 0.02, ΔRMSEA < 0.015 and TLI and RMSEA values were as good for scalar invariance compared to configural invariance, offering support for scalar invariance. Likewise, indexes of fit for configural invariance at Time 2 were χ^2^(988) = 5656.37, CFI = 0.924, TLI = 0.913, RMSEA = 0.039 (CI95% = 0.038–0.040) and scalar invariance were χ^2^(1152) = 4195.22 CFI = 0.950, TLI = 0.952, RMSEA = 0.029 (CI95% = 0.028–0.030). ΔCFI = 0.026, ΔRMSEA < 0.015 and TLI and RMSEA values were as good for scalar invariance compared to configural invariance, offering support for scalar invariance (see Table 3).

Non-standardized factor loadings were statistically invariant across the condition groups, thus only standardized factor loadings for the control group are reported. Standardized factor loadings (see Table 4) revealed all empowering and disempowering items for the control group loaded positively and strongly on their intended factor. Standardized factor loadings for empowering items ranged from 0.41 to 0.65 (*p* < 0.001) (Time 1) and 0.53 to 0.70 (*p* < 0.001) (Time 2) on the empowering factor (see Table 4) and –0.12 to 0.21 (Time 1) and –0.13 to 0.19 (Time 2) on the non-intended disempowering factor. The standardized factor loadings for the disempowering items ranged from 0.18 to 0.63 (*p* < 0.001) (Time 1) and 0.20 to 0.73 (*p* < 0.001) (Time 2) on the disempowering factor and –0.16 to 0.35 (Time 1) and –0.11 to 0.40 (*p* < 0.001) (Time 2) on the non-intended empowering factor. Item 15 loaded positively and more strongly onto the empowering compared to the disempowering factor.

For the non-reference (intervention) group, standardized factor loadings for empowering items ranged from 0.41 to 0.65 (*p* < 0.001) (Time 1) and 0.53 to 0.65 (*p* < 0.001) (Time 2) on the empowering factor and –0.14 to 0.20 (Time 1 and 2) on the disempowering factor. The standardized factor loadings for the disempowering items ranged from 0.20 to 0.61 (*p* < 0.001) (Time 1) and 0.21 to 0.71 (*p* < 0.001) (Time 2) on the disempowering factor and –0.14 to 0.34 (Time 1) and –0.10 to 0.37 (Time 2) on the empowering factor. Again, item 15 loaded positively and more strongly onto the empowering compared to the disempowering factor. As the majority of factor loadings for items on their intended factor were above 0.50, indirect support for the internal consistency of the empowering and disempowering latent variables across conditions is provided.

Finally, the correlation between the empowering and disempowering factor was –0.48 (*p* < 0.001) in the control condition and –0.40 (*p* < 0.001) in the experimental condition at Time 1. At Time 2, the correlation was –0.58 (*p* < 0.001) in the control condition and –0.53 (*p* < 0.001) in the experimental condition.

## Discussion

The purpose of this study was to further contribute to the development and validation process of the EDMCQ-C (Appleton et al., 2016) by exploring measurement invariance of a two-factor model across five languages, longitudinally, and across intervention groups in a multi-national sample of young footballer players.

In testing for invariance, total or partial scalar invariance is required to ensure future comparisons of latent mean scores (across the groups and over time) are meaningful. ESEM analyses indicated that the two-factor model tested in this study showed partial invariance across language and full scalar invariance across time and experimental condition groups. Moreover, the analyses confirmed that the majority (33 of 34) of items from the EDMCQ-C loaded positively onto their intended factor (with lower scores on the non-intended factor). Taken together, the invariance findings and factor loadings reported in this study provide additional evidence that the EDMCQ-C provides a sound measure of the array of coaching strategies central to Duda (2013) theory-informed model of the motivational climate. The questionnaire can also be used with confidence to provide meaningful comparisons of latent mean empowering and disempowering climate values in the sample of junior participants recruited in the PAPA project.

Item 15 (“My coach only allowed something we like to do at the end of training if players had done well during the session”) was the only item that failed to load as hypothesized across all tests of invariance in this study. Item 15 attempts to capture a controlling reward that coaches may employ during training, and loaded most strongly on the empowering factor across all analyses. This finding is consistent with the ESEM findings reported by Appleton et al. (2016) and Milton et al. (2018). Thus, it seems that junior athletes in the PAPA project may not interpret this particular coaching strategy as disempowering as intended by the scale’s authors. Rather, the young footballers seem to perceive that being allowed to do a favorite activity at the end of a training session on those occasions when they had done well in the session is an empowering coaching strategy.

There are a number of reasons that item 15 may load most strongly onto the empowering factor in this study. First, doing something “we like to do at the end of training” is most likely an activity (or activities) that the players enjoy, and thus creates the perception of a positive coaching strategy. Second, empowering coaching strategies are considered key predictors of the satisfaction of athletes’ basic psychological needs (Duda, 2013), including feelings of competence. It may be that the young footballers perceive the reward emphasized by item 15 as recognition for demonstrating sufficient level of performance (i.e., doing well), which may subsequently contribute to them feeling more competent. Finally, the “done well during the session” aspect of the item may have been interpreted by the athletes in a task-involving manner, reflecting their personal development, skill mastery and application of effort. Based on the findings reported here (as well as Appleton et al., 2016), we recommend that additional studies utilizing the data from the PAPA project proceed without the inclusion of item 15 from the EDMCQ-C. We also recommend that future research employing the EDMCQ should examine whether item15 cross-loads (more strongly) onto the empowering (compared to the disempowering) factor using data provided by junior sport participants from alternative (non-PAPA) samples and countries.

Although we found partial scalar invariance across languages, there were also non-invariant thresholds of nine items in this particular analyses. As highlighted above, non-invariant thresholds do not prevent a researcher from making meaningful comparisons of latent mean scores. However, a closer inspection of the results for eight of the nine items with non-invariant thresholds revealed differences in the proportion of athletes from each country for the less extreme responses (i.e., “disagree,” “neutral,” and “agree”). For example, regarding item three (“My coach gave players choices and options”), the analyses revealed that a larger proportion of the athletes in France responded “strongly disagree” and “disagree,” compared to athletes speaking the four other languages. This particular finding suggests that French speaking young footballers are given (or perceived to be given) fewer choices and options by their coaches during training and competition. Other studies (e.g., Tessier et al., 2013; Smith et al., 2016) assessing empowering and disempowering coach-created motivational climates have also revealed that French-speaking coaches provide lower levels of autonomy-supportive coaching compared to English, Greek and Spanish speaking athletes. The current study helps clarify the findings reported by Tessier et al. (2013) and Smith et al. (2016) by suggesting the differences in autonomy-support may lie in the lower choice and options provided by French speaking coaches. Conversations between the researchers and the participants during the data collection in the PAPA project provide an anecdotal explanation for this finding. Specifically, some of the French-speaking coaches perceived that giving their players choices and options was a sign of weakness and lack of authority, and the players reported that they did not understand choices their coaches could offer. Given that offering meaningful choices and attractive options is a key facet of an empowering climate, future intervention work with French-speaking coaches may wish to target this particular empowering strategy.

Regarding item 31 (“My coach tried to interfere in aspect of players’ lives outside of football”), the analyses revealed that Spanish speaking players’ responses were more optimistic (i.e., more players responding “strongly disagree”) compared to players from the other countries (albeit more athletes from the other countries disagreed than agreed with this statement). Why more Spanish speaking players responded strongly disagreed is not clear in the current study. It would be interesting to determine in future research whether this particular finding emerges when examining language invariance of the EDMCQ-C with athletes from other sports and competitive levels. Consistent support for this finding across other sports would suggest coaches in Spain are less disempowering with respect to interfering in their athletes’ lives outside of sport compared to coaches in other countries.

While the findings of this study are informative regarding the psychometric properties of the EDMCQ-C, we also recognize some limitations. For example, we were unable to test for gender invariance due to a gender imbalance in the sample and because in some cases, boys and girls were nested within the same team (i.e., they played for the same team). Future research may wish to test the gender invariance of the EDMCQ when completed by young athletes, and well as replicating the analyses reported in this study with a more diverse sample of athletes (e.g., older athletes, range of sports, more diverse range of languages). Consistent with recommendations (Muthén and Muthén, 1998–2015) for categorical-ordered data, we made the decision to constrain the factor loadings and thresholds in tandem, freeing the latter when testing partial (language) invariance. As a result, we did not test for factor loading invariance. We made this decision, as per previous studies (e.g., Myers et al., 2011; Viladrich et al., 2013), due to the complexities associated with managing factor loading non-invariance attributable to one language on one item in an ESEM. Moreover, with categorical data, the factor loadings and thresholds are not independent as both contribute to the item characteristic function. Based on the difficulties reported by Appleton et al. (2016) in modeling the hierarchical structure of the EDMCQ-C, we also decided to limit our analyses to a two-factor model that reflected the overall empowering and disempowering features of the coach-created motivational climate. However, if advances are made in refining the content of the EDMCQ-C to enable the successful modeling of the scale’s hierarchical nature, future research will be needed to re-examine the scale’s invariance. Finally, future research may wish to include interactional invariance tests (e.g., across countries, experimental conditions, time points, gender), which was not possible in this study due to the complexity of the analyses and the nature of our data (missing data, sample size and skewness).

## Conclusion

Collectively, the results from this study provide further evidence on the psychometric properties of the EDMCQ-C as a measure of the empowering and disempowering features of the coach-created motivational climate in youth sport. Overall, the findings suggest the scale (minus item 15) may be used to provide meaningful latent mean comparisons (Marsh et al., 2013) across the five targeted languages, time, and experimental groups when utilizing the data from the PAPA project. This is especially important given the PAPA project attempted to help coaches create empowering (and reduced disempowering) motivational climates in youth sport. The findings reported in this study should also increase researchers’ confidence in using the EDMCQ-C as a measure of athletes’ perceptions of empowering and disempowering coach-created motivational climate in future research. Such research could use the EDMCQ-C to test theory-informed process models (see Duda and Appleton, 2016; Duda et al., 2018) that include the two climate factors proposed in this study, and such research would also contribute to the nomological validity of the EDMCQ-C.

## Data availability statement

The raw data supporting the conclusions of this article will be made available by the authors, without undue reservation.

## Ethics statement

Ethical approval was granted by the Universities of each team of researchers working on the PAPA project. Prior to collecting data, parents had the opportunity to opt-out their son and/or daughter from the project. Participants were fully informed about the study prior to it taking place and a parental written opt-out form was used in accordance with national legislation and the institutional requirements.

## Author contributions

PA and JD: conceived the study. PA, EQ, and JD: designed the original questionnaire. IB and LG-G: designed the Spanish version of the questionnaire. PS and J-PH: designed the French version of the questionnaire. AP: designed the Greek version of the questionnaire. YO: designed the Norwegian version of the questionnaire. JD, IB, AP, HH, PS, J-PH, YO, BW, and OS: funding acquisition. JD: supervised the manuscript. PA, EQ, LG-G, AP, HH, IB, YR, PS, J-PH, YO, BW, OS, and JD: collected the data. PA and CV: analyzed the data. PA, CV, and JD: interpreted the results of the research. CV: designed the figures and tables. PA and CV: wrote and edited the manuscript. All authors provided critical revisions and the formal analysis on the successive drafts and approved the manuscript in its final form.
